# Inhibition of microRNA-29a alleviates hyperoxia-induced bronchopulmonary dysplasia in neonatal mice via upregulation of GAB1

**DOI:** 10.1186/s10020-019-0127-9

**Published:** 2019-12-31

**Authors:** Yu Hu, Liang Xie, Jing Yu, Hongling Fu, Dan Zhou, Hanmin Liu

**Affiliations:** 1 West China School of Medicine, Sichuan University, Chengdu, 610041 People’s Republic of China; 2 Mianyang Central Hospital, Department of Pediatrics, Mianyang, People’s Republic of China; 3 Mianyang, 621000 People’s Republic of China; 4 The Vascular Remodeling and Developmental Defects Research Unit, West China Institute of Women and Children’s Health, West China Second University Hospital, Sichuan University, Chengdu, 610041 People’s Republic of China; 5 Department of Pediatric Respiratory, West China Second University Hospital, Sichuan University, No. 17, Renmin South Road, Chengdu, 610041 Sichuan Province People’s Republic of China

**Keywords:** Bronchopulmonary dysplasia, MicroRNA-29a, GAB1, Apoptosis, Hyperoxia, MLE-12

## Abstract

**Background:**

The main features of bronchopulmonary dysplasia (BPD) are alveolar simplification, pulmonary growth arrest, and abnormal lung function. Multiple studies have highlighted microRNA-29 (miR-29) as a potential biomarker for lung diseases and cancers. Upregulation of miR-29a has been known to downregulate GRB2-associated-binding protein 1 (GAB1), which is often highly expressed in the lung. The current study was designed to investigate the potential role of miR-29a in hyperoxia-induced BPD by targeting GAB1 in a neonatal mouse model.

**Methods:**

The expression of miR-29a and GAB1 in lung tissues of neonatal mice with hyperoxia-induced BPD and mouse alveolar epithelial cells (MLE-12) was determined using RT-qPCR and western blot analysis. Subsequently, the relationship between miR-29a and GAB1 was verified using in silico analysis. In order to assess the effects of miR-29a or GAB1 on BPD, the pathological characteristics of alveoli, as well as proliferation and apoptosis of cells were measured through gain- and loss-of-function studies.

**Results:**

Upregulation of miR-29a and downregulation of GAB1 were evident in both lung tissues and MLE-12 cells following BPD modeling. GAB1 was a direct target gene of miR-29a. Inhibition of miR-29a and overexpression of GAB1 were shown to alleviate lung injury, promote cell proliferation and inhibit apoptosis but reduce chord length in lung tissues of neonatal mice following hyperoxia-induced BPD modeling.

**Conclusion:**

Altogether, down-regulation of miR-29a can potentially elevate GAB1 expression, reducing cell apoptosis and stimulating proliferation, ultimately retarding the development of BPD in mice. This study highlights the potential of a promising new target for preventing BPD.

## Background

In 2016, neonatal preterm birth complication ranked as one of the three leading causes of mortality worldwide in children under 5 years of age (Collaborators 2017). A chronic lung disorder of preterm birth, bronchopulmonary dysplasia (BPD) is triggered by the disturbances in physiologic lung development (Shahzad et al. 2016). BPD is predominantly characterized by simplified alveolar structure, arrested lung growth, impaired vascular development, and abnormal pulmonary function (Michael et al. 2018). It is documented that BPD leads to a remarkable morbidity and mortality among preterm infants (Pasha et al. 2018). Consequently, It was shown that it is important to investigate the molecular mechanisms underlying BPD in order to identify more effective BPD treatment methods.

MicroRNAs (miRNAs) are known to play a role in the pathogenesis of various human diseases due to their regulatory functions in cell development, differentiation, proliferation, in addition to their cell type-specific functions (Chiofalo et al. 2017). MiR-29a, a member of the miR-29 family, is aberrantly expressed in a variety of tumors and affects several pathological processes including tumor growth and apoptosis (Fiserova et al. 2015). While involvement of some miRNAs in the key steps of early lung development is well known, the crucial role of miR-29 family in BPD has recently attracted a lot of attention (Nardiello and Morty 2016). It was shown that miR-29a regulates non-small cell lung cancer (NSCLC) cell invasion, migration, and proliferation (Li et al. 2017). Furthermore, Dong et al. demonstrated prominently increased levels of miR-29a in the lung tissues in BPD mouse models (Dong et al. 2012). In a recent study, inhibition of miR-29a induces upregulation of GRB2-associated-binding protein 1 (GAB1) to protect human osteoblasts from hydrogen peroxide (Ruan et al. 2018). GAB1 belongs to the GAB adaptor family, and silencing of GAB1 might deregulate pulmonary surfactants and enhance pulmonary susceptibility to inflammatory responses (Wang et al. 2016). Also, GAB1 has been suggested to be a novel ideal target for controlling epidermal growth factor receptor mutant lung cancer (Takeuchi et al. 2012). It has been reported that rs1397529 in GAB1 is negatively associated with the risk of lung cancer, and could serve as a novel biomarker for lung cancer (Li et al. 2017). In the present study, we aim to investigate the possible regulatory effects associated with miR-29a on lung cell apoptosis and proliferation in a neonatal mouse model of hyperoxia-induced BPD, along with the underlying mechanism associated with GAB1.

## Materials and methods

### Ethics statement

The current study was performed with the approval of the Ethics Committee of West China Second University Hospital, Sichuan University. All animal procedures were conducted in accordance with the Guide for the Care and Use of Laboratory Animals published by National Institutes of Health.

### Establishment of hyperoxia-induced BPD mouse model

Fifteen female specific pathogen free Kunming (KM) mice with the same gestational weeks (provided by the Experimental Animal Center of West China Second University Hospital, Sichuan University) were used for spontaneous delivery. Then, 160 neonatal mice (male or female, weighing 3.78 ± 0.41 g) were randomly selected and assigned into the room air (RA) group (*n* = 35) and the BPD group (hyperoxia-induced BPD, *n* = 120). Prior to hyperoxia treatment, the mice were subcutaneously injected with 5 μL adenovirus (1 × 10^9^ pfu/100 μL) or miR-29a antagomir and its control (20 nM). The synthesis of adenovirus expression vector overexpressing (oe)-GAB1 (pAAV-CAG-RFP-GAB1) and miR-29a antagomir was conducted by Shanghai GenePharma Co. Ltd. (Shanghai, China). Next, the neonatal mice with hyperoxia-induced BPD received an injection of adenovirus-packaged oe-GAB1 vector, miR-29a antagomir, and their corresponding negative control (NC). Afterwards, both neonatal and mother mice were placed in a sealed polypropylene cage and subjected to hyperoxia treatment by continuous oxygen (5 L/min) input into the tank with FiO2 being maintained > 90%, which was measured by an oxygen analyzer, temperature at 22–27 °C and humidity between 50 and 70%. Soda lime was used to absorb CO_2_, and color-changing silica gel was used to absorb water. Neonatal mice in the RA group were placed in a standard breeding cabin with air as the inhalation gas, and other conditions were same as those for neonatal mice in the BPD group. On alternate days, the mother mice were exchanged between RA and hyperoxia using lactation dams in order to prevent the mother mice from dying due to hyperoxia exposure and to ensure that they had adequate nutrition. During the experiment, the weight and activity of the mother mice were found normal. For the BPD model, neonatal mice were exposed to hyperoxia from postnatal day 1 to 4, then allowed to recover in RA for the following 10 days and euthanized on postnatal day 14, as previously described (Syed et al. 2017). The survival rate and general state of neonatal mice were observed and recorded every day. The successful establishment of hyperoxia-induced BPD mouse model was confirmed by hematoxylin-eosin (HE) staining, counting radial alveolar counts (RAC), recording alveolar chord length and measuring alveolar cross-sectional area (the details are stated in the following text). On day 0, day 3, day 5, and day 7 after birth, 5 randomly selected mice in each group were intraperitoneally euthanized by intraperitoneal injection of 5% pentobarbital sodium, followed by extracting lung tissue to measure miR-29a expression. The remaining mice, on day 14 after birth, were injected with 5% pentobarbital sodium for anesthesia and euthanized thereafter. The trachea of mice was intubated to maintain the pulmonary inflated state, after which the chest was opened followed by exsanguination. The left bronchus was ligated and rapidly injected with 4% paraformaldehyde until the left lung was inflated at 25 cm H_2_O airway pressure. Lung tissues were then extracted in a rapid manner and the left lung was fixed completely with 10% neutral formalin before paraffin sectioning. Subsequently, the right lung was extracted, frozen in liquid nitrogen and then stored in a − 80 °C freezer.

### HE staining

The left lung tissues of the aforementioned mice were collected, embedded with paraffin in a conventional manner, and cut into serial sections. The sections were dewaxed, hydrated and subjected to HE staining followed by mounting and observation under an optical microscope. Two tissue sections of each group were selected at each time point, and 5 fields of each section were observed under an optical microscope for morphological analyses. A vertical line from the center of the respiratory bronchiole to the distal pleura was used to count the number of alveoli on the line for average calculation of RAC. Image-Pro Plus 6.0 image analysis software was used to measure alveolar chord length. According to the average chord length, the alveolar size and alveolar cross-sectional area were estimated (alveolar area/pulmonary septal area ratio [A/S] was measured by Image Analysis System [Universal Imaging Corporation, West Chester, Pa., USA]). Alveolar area and pulmonary septal area were determined in 5 randomly selected visual fields from each section at different time points in each group.

### Immunohistochemistry

The paraffin-embedded left lung tissue sections of neonatal mice were blocked with normal goat serum working solution (Shanghai Sangon Biotechnology Co. Ltd., Shanghai, China) at room temperature for 20 min, and then probed overnight with antibodies (Abcam Inc., Cambridge, UK) as follows: rabbit polyclonal antibody to Ki67 (1: 50, ab833), rabbit polyclonal antibody to GAB1 (1: 50, ab59362) and rabbit polyclonal antibody to CD31 (1: 50, ab28364) at 4 °C. Next, the sections were then re-probed with biotin-labeled goat anti-rabbit immunoglobulin G (IgG) (1: 1000, ab6721, Abcam Inc., Cambridge, MA, USA) for 30 min followed by incubation in streptavidin-biotin complex (Vector Labs, Burlingame, CA, USA) at 37 °C for 30 min. The sections were subsequently developed using diaminobenzidine (DAB) working solution (Sigma-Aldrich Chemical Company, St Louis, MO, USA) for 6 min, counterstained with hematoxylin for 30 s, and dehydrated with gradient ethanol (70, 80, 90, 95%) and absolute ethanol for 2 min, respectively. Finally, the sections were immersed twice with xylene for 5 min, mounted with neutral resin, and observed under an optical microscope (Olympus Optical Co., Ltd., Tokyo, Japan). The cells with the cytoplasm stained brownish-yellow were considered as positively stained cells. Under the optical microscope, 5 high power microscope fields were randomly selected, and the average positively stained cells was calculated. Then 100 cells were counted in each field, and positive rate was calculated as the percentage of the number of positively stained cells to total cells.

### TdT-mediated dUTP-biotin nick end-labeling (TUNEL) staining

The apoptosis in the left lung tissue sections was detected according to the instructions of a TUNEL kit (Roche, Basel, Switzerland). Briefly, the sections of neonatal mice lung tissues were added with 50 μL TUNEL reaction solution (enzyme concentration solution and labeling solution, 1: 9) and reacted for 50 min. Then, the sections were added with 50 μL peroxisome and incubated for 30 min at 37 °C, followed by color development with 100 μL DAB working solution for 10 min. Next, the sections were counterstained with hematoxylin for 3 s, sealed with neutral resin, and observed under a microscope of high magnification. At last, the apoptotic cells were counted in 5 randomly selected fields from each group, and the apoptosis rate was calculated using the formula: apoptosis rate = (the number of apoptotic cells / the number of total cells) × 100%.

### Cell culture and transfection

Mouse alveolar epithelial cells (MLE-12) (American Type Culture Collection [ATCC], Manassas, VA, USA) were cultured in Dulbecco’s Modified Eagle’s medium (DMEM; 10,099,141, Gibco, Grand Island, NY, USA) supplemented with 10% fetal bovine serum (FBS). The cells were then subcultured in an incubator (thromo3111, Beisheng Medical Instrument Co., Ltd., Jinan, Shandong, China) with 5% CO_2_ at 37 °C for 3–4 days, with the culture medium being replaced every 2 days. Subsequently, the MLE-12 cells were collected following conventional detachment and then subjected to transfection of following plasmids (all purchased from Shanghai GenePharma Co., Ltd., Shanghai, China): miR-29a mimic, combination of hyperoxia and miR-29a inhibitor, and their corresponding controls. The culture medium was replaced by culture medium without antibiotics and 10% FBS one day prior to cell transfection. When the cell density reached 70–80%, the cell transfection was carried out in accordance with the instructions of Lipofectamine 2000 (Invitrogen, Carlsbad, CA, USA). After transfection for 48 h, the cells were harvested for subsequent experiments.

### Dual luciferase reporter gene assay

TargetScan (http://www.targetscan.org/vert_71/) was used to predict the potential target genes of miR-29a, and GAB1 was predicted as one of the candidate genes. To further validate the relationship between miR-29a and GAB1, synthetic 3’untranslated region (3’UTR) fragments of GAB1 (GAB1-wild type [Wt]) were introduced into pMIR-reporter plasmids (Huayueyang Biotechnology Co., Ltd., Beijing, China) via endonuclease sites SpeI and Hind III. Afterwards, the target fragments were inserted into pMIR-reporter plasmids using T4 DNA ligase. Therefore, the pMIR-reporter plasmids with Wt GAB1 (pMIR-GAB1-Wt) and GAB1 mutant (Mut) at the putative miR-29a binding sites (pMIR-GAB1-Mut) were designed. The pMIR-GAB1-Wt plasmids and pMIR-GAB1-Mut plasmids were respectively co-transfected with miR-29a mimic or mimic-NC using Lipofectamine 2000 into the cells (Beinuo Biotechnology Co., Ltd., Beijing, China). The cells were transfected with pRL-TK plasmids, which was used as reference. After transfection for 48 h, the cells were collected and lysed. Dual-Luciferase Reporter assay kit (K801–200, Biovision, Milpitas, CA, USA) and Glomax 20/20 luminometer fluorescence detector (Promega, Madison, WI, USA) were applied for luciferase activity detection. The experiment was repeated 3 times independently.

### Reverse-transcription quantitative polymerase chain reaction (RT-qPCR)

Total RNA in tissues and cells were extracted using the Trizol kit (16,096,020, Thermo Fisher Scientific Inc., Waltham, MA, USA), and then reversely transcribed into complementary DNA (cDNA) using the Reverse Transcription kit (QPG-040~QPG-043, Shanghai GenePharma Co., Ltd., Shanghai, China). All the primers (Table 1) were synthesized by TaKaRa (Dalian, Liaoning, China). Based on the previous literature (Li et al. 2017), the quantitative analysis of mRNA expression was conducted according to the instructions of TaqMan MicroRNA Assay kit (Applied Biosystems, Foster City, CA, USA). U6 was used to standardize miR-29a expression while the expression of remaining genes was standardized against glyceraldehyde 3-phosphate dehydrogenase (GAPDH) levels. 2^-ΔΔCt^ represented the ratio of the expression of the target gene in the experiment group and the control group. The following formula was used for calculation: ΔΔCT = ΔCt _experimental group_ - ΔCt _control group_, where ΔCt = Ct _target gene_ - Ct _control gene_. The experiment was repeated 3 times independently. he miR-29a expression was standardized using U6, and the expression of remaining genes was standardized using glyceraldehyde 3-phosphate dehydrogenase (GAPDH).

**Table 1:** Primer sequences of RT-qPCR

Gene	Sequence
miR-29a	F: 5′-CTGATTTCTTTTGGTGTTCAG-3’
	R: 5′-AACCGATTTCAGATGGTGC-3’
GAB1	F: 5′-GAAGTTGAAGCGTTATGCGTG-3’
	R: 5′-TCCAGGACATCCGGGTCTC-3’
U6	F: 5′-TCCGACGCCGCCATCTCTA-3’
	R: 5′-TATCGCACATTAAGCCTCT-3’
GAPDH	F: 5′-AGGGCATCTTGGGCTACAC-3’
	R: 5′-TGGTCCAGGGTTTCTTACTCC-3’

Note: RT-qPCR, reverse transcription quantitative polymerase chain reaction; miR-29a, microRNA-29a; GAB1, grb2-associated-binding protein 1; GAPDH, glyceraldehyde 3-phosphate dehydrogenase; F, forward; R, reverse

### Western blot analysis

Western blot analysis was performed as described previously (Rojo et al. 2012). Briefly, total protein in tissues and cells was extracted using radio-immunoprecipitation assay lysis buffer (C0481, Sigma-Aldrich Chemical Company, St Louis, MO, USA). Protein quantification was performed according to the instructions of a bicinchoninic acid (BCA) kit. The proteins separated by sodium dodecyl sulfate-polyacrylamide gel electrophoresis (SDS-PAGE) were transferred onto the polyvinylidene fluoride (PVDF) membrane. The membrane was blocked with Tris-buffered saline Tween-20 (TBST) buffer containing 5% skim milk powder for 1 h and probed overnight at 4 °C with primary rabbit polyclonal antibodies (Abcam Inc., Cambridge, MA, USA) against GAB1 (1: 500, ab59362), Bcl-2-associated X protein (Bax; 1: 1000, ab199677), B-cell lymphoma 2 (Bcl-2; 1: 1000, ab196495), Caspase-3 (1: 200, ab4051), and β-actin (1: 1000, ab8227). Then the membrane was incubated with biotin-labeled goat anti-rabbit IgG (1: 2000, ab6721) for 1 h at room temperature. Finally, the protein on the membrane was visualized using enhanced chemiluminescence (ECL) reagent and band intensities were quantified using GeneTools (Syngene, San Diego, CA, USA). The relative protein expression was calculated as the ratio of the band intensities of the target protein band to β-actin. The experiment was repeated 3 times independently.

### Statistical analysis

All experimental data were analyzed using SPSS 21.0 software (IBM Corp., Armonk, NY, USA). All data were subjected to test of normal distribution and variance homogeneity, and measurement data were expressed as mean ± standard deviation. Normally distributed data with homogeneous variance between two groups were compared using unpaired *t*-test while Welch’s t-test was used for unequal variances. Repeated-measures analysis of variance (ANOVA) was utilized for comparison of data at different time points. Data among multiple groups were analyzed by ANOVA, followed by a Tukey multiple comparison test. The rank-sum test was performed for comparison of data with skewed-distributed. *p* < 0.05 was considered to be indicative of statistical significance.

## Results

### Successful establishment of BPD mouse model

**Fig. 1:**
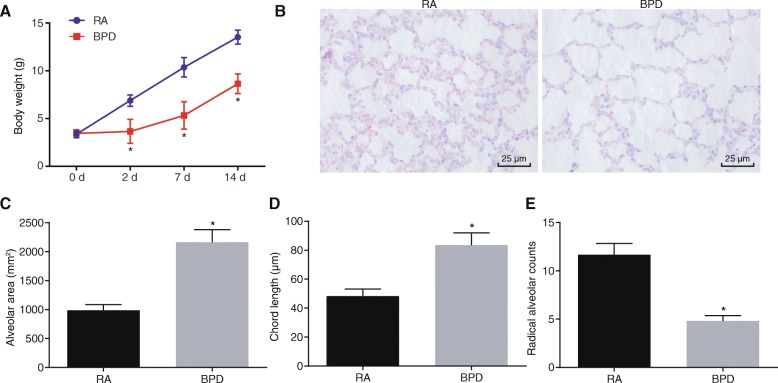
Hyperoxia-induced BPD mouse models are successfully established. A, Body weight of neonatal mice under exposure of RA or hyperoxia. B, HE staining of lung tissues of neonatal mice under exposure of RA or hyperoxia (× 400). C, Alveolar cavity area in lung tissues in neonatal mice under exposure of RA or hyperoxia. D, Alveolar chord length in lung tissues in neonatal mice under exposure of RA or hyperoxia. E, RAC in lung tissues of neonatal mice under exposure of RA or hyperoxia. *N* = 15. ^*^ *p* < 0.05 vs. the neonatal mice under RA exposure (the RA group). Measurement data were expressed as mean ± standard deviation. Data comparison between two groups was examined by unpaired *t*-test. Comparison of data at different time points was examined by repeated-measures analysis of variance. BPD, bronchopulmonary dysplasia; RA, room air; HE, hematoxylin-eosin; RAC, radial alveolar counts

BPD mouse models were established through exposing neonatal mice to hyperoxia. In order to confirm the successful establishment of BPD mouse models, the general state of neonatal mice under exposure of RA or hyperoxia were observed. Neonatal mice under exposure of RA exhibited good appetite, nice physical activity, strong and plump figure, and bright fur. However, neonatal mice exposed to hyperoxia responded poorly, with reduced autonomic activity, dull fur, and mild breathing difficulty from oxygen. The body weight of neonatal mice was recorded, and the data showed that body weight of mice began to decrease significantly the 3rd day after hyperoxia exposure (i.e. postnatal day 7) compared with those exposed to RA (*p* < 0.05; Fig. 1A), suggesting that the mice would lose weight under hyperoxia exposure. At the 10th day following hyperoxia treatment (postnatal day 14), lung tissues of neonatal mice those were exposed to RA or hyperoxia were collected for HE staining analysis in order to observe histopathological changes. Lung tissues of neonatal mice exposed to RA exhibited normal and clear alveolar structure, a large number of alveoli with uniform size and a small alveolar cavity (*p* < 0.05). While the lung tissues of hyperoxia-treated neonatal mice showed disordered alveolar structure, reduced number of alveoli, significantly increased alveolar cavity with a simple structure, and thinner alveolar space with appearance of alveoli fusion (pulmonary bulla: it refers to the pressure increase in the alveolar space due to various reasons, and then the alveolar wall ruptures and fuses with each other, consequently the balloon-containing cavity being formed in the lung tissue) (*p* < 0.05; Fig. 1B). Additionally, the alveolar cavity area and alveolar chord length of lung tissues notably increased in neonatal mice exposed to hyperoxia while RAC decreased in comparison with those exposed to RA (*p* < 0.05; Fig. 1C-E). These results were indicative of successful establishment of mouse model.

### Downregulation of miR-29a prevents hyperoxia-induced BPD

**Fig. 2:**
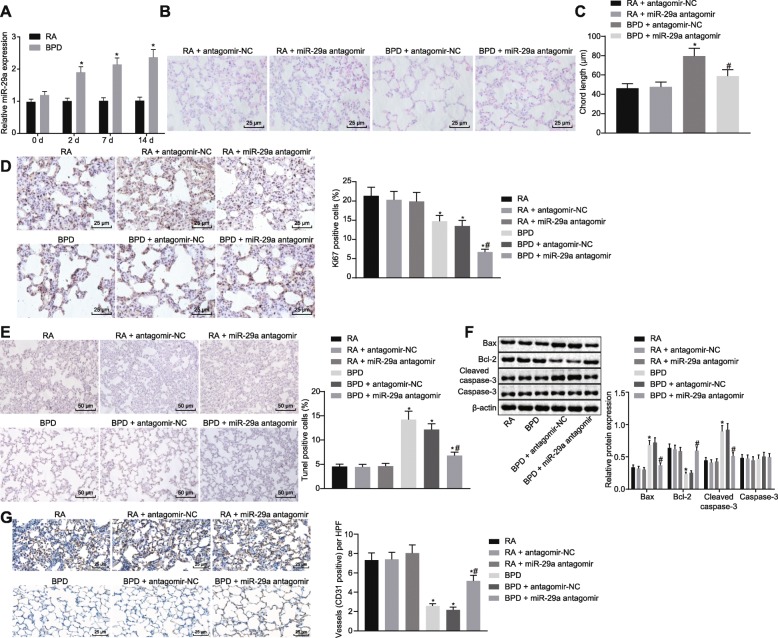
Inhibition of miR-29a impedes hyperoxia-induced BPD in neonatal mice. A, The expression of miR-29a in lung tissues of neonatal mice under exposure of RA or hyperoxia determined by RT-qPCR. ^*^ *p* < 0.05 vs. neonatal mice under exposure of RA (the RA group). B, HE staining analysis of lung tissues in each group (× 400). C, Alveolar chord length of lung tissues in each group. D, Positive expression rate of Ki67 protein in lung tissues of each group determined by immunohistochemistry (× 400). E, Cell apoptosis in lung tissues of each group assessed by TUNEL staining (× 200). F, Western blot analysis of apoptosis-related proteins (Bax, Caspase-3 and Bcl-2) in lung tissues of each group. G, Positive expression rate of CD31 protein in lung tissues of each group determined by immunohistochemistry (× 400). In panel C-F, ^*^ *p* < 0.05 vs. neonatal mice under exposure of RA (the RA group). ^#^ *p* < 0.05 vs. neonatal mice with hyperoxia-induced BPD receiving antagomir-NC (the BPD + antagomir-NC group). *N* = 15. Measurement data were expressed as mean ± standard error. Data comparison between two groups was examined by unpaired *t*-test. miR-29a, microRNA-29a; BPD, bronchopulmonary dysplasia; RA, room air; Bax, Bcl-2-associated X protein; Bcl-2, B-cell lymphoma 2; RT-qPCR, reverse-transcription quantitative polymerase chain reaction; HE, hematoxylin-eosin; RAC, radial alveolar counts; TUNEL, TdT-mediated dUTP-biotin nick end-labeling; NC, negative control

MiR-29a has been reported to be upregulated in BPD (Dong et al. 2012), but its specific role and the underlying mechanism were still unknown. In order to confirm the expression of miR-29a in BPD, RT-qPCR was used to determine miR-29a expression in lung tissues of neonatal mice. As depicted in Fig. 2A, miR-29a expression was much higher in lung tissues of neonatal mice under hyperoxia exposure from postnatal day 2 to 14 than in neonatal mice under RA exposure (*p* < 0.05).

In order to study the effects of miR-29a downregulation on BPD, the neonatal mice with hyperoxia-induced BPD received an injection of miR-29a antagomir or antagomir-NC. Initially, HE staining was performed to observe the histopathological changes in the lung tissues. The results showed that after receiving an injection of miR-29a antagomir, the neonatal mice with hyperoxia-induced BPD exhibited increased number of alveoli in lung tissues accompanied with significantly reduced alveolar space and decreased alveoli fusion, when compared with those treated with antagomir-NC (*p* < 0.05; Fig. 2B). Subsequently, the measurement of alveolar chord length, assessment of positive expression of Ki67 and CD31 proteins by immunohistochemistry, and detection of lung cell apoptosis by TUNEL staining was performed to further evaluate the effect of miR-29a inhibition on BPD. The positive expression rate of Ki67 and CD31 proteins displayed a notable decline in lung tissues of neonatal mice with hyperoxia-induced BPD with an enhancement in alveolar chord length and cell apoptosis compared with those under RA exposure (*p* < 0.05). There was upward trend in alveolar chord length and cell apoptosis while downward trend in positive expression rate of Ki67 and CD31 proteins in BPD mice treated with miR-29a antagomir as compared to the mice exposed to RA following miR-29a antagomir treatment (*p* < 0.05). However, the tendency of these parameters was opposite in neonatal mice with hyperoxia-induced BPD after receiving miR-29a antagomir injection compared with those receiving antagomir-NC (*p* < 0.05; Fig. 2C-E & G). Furthermore, western blot analysis was performed to determine the expression of apoptosis-related proteins (Bax, Caspase-3 and Bcl-2). The results revealed that the lung tissues of neonatal mice with hyperoxia-induced BPD showed increased Bax and Caspase-3 expression yet decreased Bcl-2 expression in contrast to neonatal mice under RA exposure (*p* < 0.05). The expression of Bax and Caspase-3 was upregulated while Bcl-2 expression was downregulated in BPD mice treated with miR-29a antagomir when compared to the mice exposed to RA following miR-29a antagomir treatment (*p* < 0.05). Injection of miR-29a antagomir into neonatal mice with hyperoxia-induced BPD resulted in an opposite trend compared with those treated with antagomir-NC (*p* < 0.05) (Fig. 2F). The aforementioned data supported that downregulation of miR-29a led to reduced apoptosis and thus alleviated the disease process of BPD in neonatal mice.

### GAB1 is a target gene of miR-29a

**Fig. 3:**
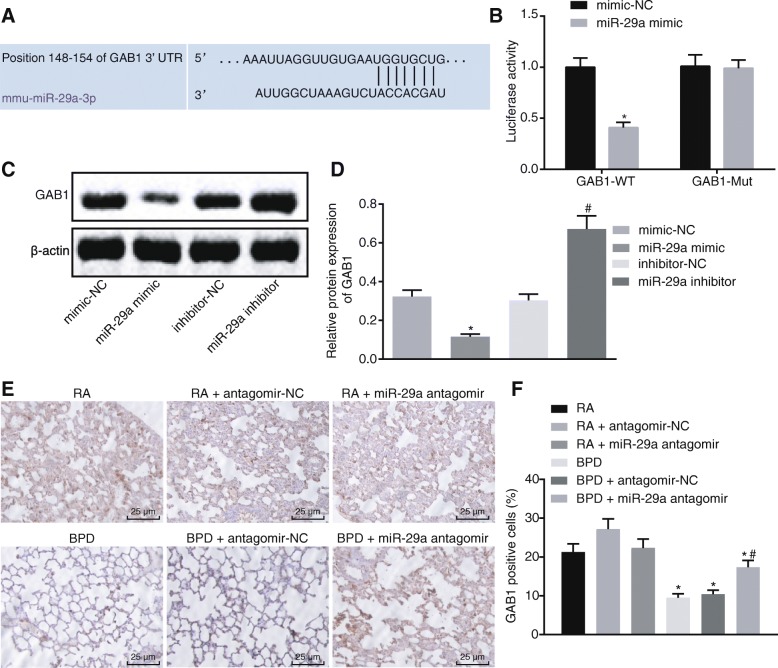
GAB1 is a target gene of miR-29a. A, Predicted binding site between miR-29a and GAB1. B, The binding of miR-29a to GAB1 verified by dual-luciferase reporter gene assay. ^*^ *p* < 0.05 vs. the cells co-transfected with mimic-NC and pMIR-GAB1-Wt plasmids or pMIR-GAB1-Mut plasmids (the mimic-NC group). C&D, Western blot analysis of GAB1 protein in MLE-12 cells of each group. ^*^ *p* < 0.05 vs. MLE-12 cells transfected with mimic-NC (the mimic-NC group). ^#^ *p* < 0.05 vs. MLE-12 cells delivered with inhibitor-NC (the inhibitor-NC group). E&F, the positive expression of GAB1 protein in lung tissues of each group detected by immunohistochemistry (× 400). ^*^ *p* < 0.05 vs. neonatal mice under exposure of RA (the RA group). ^#^ *p* < 0.05 vs. neonatal mice with hyperoxia-induced BPD receiving miR-29a antagomir administration (the BPD + miR-29a antagomir group). *N* = 15. Measurement data were expressed as mean ± standard error. Data comparison between two groups was examined by unpaired *t*-test. The experiment was repeated 3 times independently. miR-29a, microRNA-29a; GAB1, GRB2-associated-binding protein 1; BPD, bronchopulmonary dysplasia; RA, room air; Wt, wild type; Mut, mutant; NC, negative control

In order to examine the regulatory role of miR-29a in BPD, we performed in silico analysis using TargetScan to find potential miR-29a target genes. GAB1 was one of the predicted target genes of miR-29a (Fig. 3A). Then dual-luciferase reporter gene assay was performed on mouse alveolar epithelial cells (MLE-12) for further verification. It was observed that co-transfection of miR-29a mimic and pMIR-GAB1-Wt led to reduced luciferase activity as compared to co-transfection of mimic-NC and pMIR-GAB1-Wt (*p* < 0.05). No significant difference was observed in the luciferase activity in cells co-transfected with pMIR-GAB1-Mut and miR-29a mimic or mimic-NC (*p* > 0.05; Fig. 3B). Moreover, western blot analysis demonstrated that overexpression of miR-29a resulted in a decline in GAB1 expression (*p* < 0.05). On the contrary, inhibition of miR-29a increased GAB1 expression (*p* < 0.05; Fig. 3C-D). Likewise, the positive expression rate of GAB1 protein in lung tissues of neonatal mice detected by immunohistochemistry indicated that the positive expression rate of GAB1 protein exhibited a downregulation in neonatal mice with hyperoxia-induced BPD (*p* < 0.05). BPD mice treated with miR-29a antagomir showed a prominent increase in the positive expression rate of GAB1 protein in comparison to those treated with antagomir-NC (*p* < 0.05). Similar results were obtained in RA mice exposed to RA upon miR-29a antagomir treatment as compared to those upon antagomir-NC treatment (*p* < 0.05; Fig. 3E-F). Taken together, GAB1 expression was downregulated in lung tissues of neonatal mice with hyperoxia-induced BPD and it can be negatively regulated by miR-29a.

### Upregulation of GAB1 alleviates hyperoxia-induced BPD

**Fig. 4:**
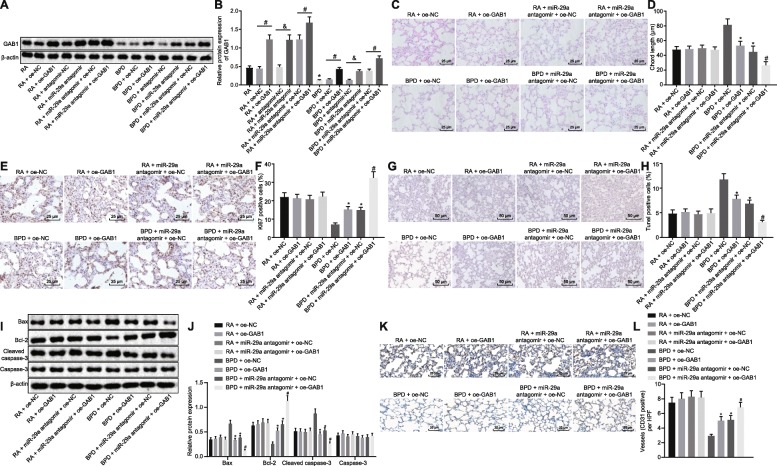
Upregulation of GAB1 ameliorates BPD in neonatal mice. A&B, Western blot analysis of GAB1 protein in lung tissues of each group. ^*^ *p* < 0.05 vs. neonatal mice under RA exposure (the RA group). ^#^ *p* < 0.05 vs. neonatal mice with hyperoxia-induced BPD receiving oe-NC vector injection (the BPD + oe-NC group). ^&^ *p* < 0.05 vs. neonatal mice with hyperoxia-induced BPD receiving antagomir-NC injection (the BPD + antagomir-NC group). C, HE staining analysis of lung tissues in each group (× 400). D, Alveolar chord length of lung tissues in each group. E&F, Positive expression level of Ki67 in lung tissues of each group determined by immunohistochemistry (× 400). G&H, Cell apoptosis in lung tissues of each group assessed by TUNEL staining (× 200). I&J, Western blot analysis of apoptosis-related proteins (Bax, Caspase-3 and Bcl-2) in lung tissues of each group. K&L, Positive expression rate of CD31 protein in lung tissues of each group determined by immunohistochemistry (× 400). In panel C-L, ^*^ *p* < 0.05 vs. neonatal mice with hyperoxia-induced BPD receiving oe-NC vector injection (the BPD + oe-NC group). ^#^ *p* < 0.05 vs. neonatal mice with hyperoxia-induced BPD receiving an injection of antagomir-NC combined with oe-NC vector (the BPD + antagomir-NC + oe-NC group). N = 15. Measurement data were expressed as mean ± standard error. Data comparison between two groups was examined by unpaired *t*-test. miR-29a, microRNA-29a; GAB1, GRB2-associated-binding protein 1; BPD, bronchopulmonary dysplasia; RA, room air; Bax, Bcl-2-associated X protein; Bcl-2, B-cell lymphoma 2; HE, hematoxylin-eosin; RAC, radial alveolar counts; TUNEL, TdT-mediated dUTP-biotin nick end-labeling; NC, negative control

GAB1 had been confirmed to be a target gene of miR-29a, and then the effects of miR-29a regulating GAB1 on BPD were subsequently investigated. Neonatal mice with hyperoxia-induced BPD received an injection of adenovirus-packaged oe-GAB1 vector or its NC. Western blot analysis demonstrated that GAB1 expression was increased in BPD mice after oe-GAB1 vector injection compared with oe-NC vector injection (*p* < 0.05). GAB1 expression was much higher in mice exposed to RA following oe-GAB1 injection than those injected with oe-NC (*p* < 0.05; Fig. 4A-B). Additionally, lung tissues of neonatal mice with hyperoxia-induced BPD receiving an injection of miR-29a antagomir exhibited increased GAB1 expression compared to those treated with antagomir-NC (*p* < 0.05). In comparison to the mice exposed to RA following antagomir-NC injection, GAB1 expression was elevated in mice exposed to RA in response to miR-29a antagomir injection (*p* < 0.05; Fig. 4A-B). These results demonstrated that miR-29a downregulation could enhance GAB1 expression in neonatal mice with hyperoxia-induced BPD.

Furthermore, HE staining was performed to observe the histopathological changes of lung tissues. As shown in Fig. 4C, after receiving an injection of oe-GAB1 vector, the neonatal mice with hyperoxia-induced BPD exhibited increased number of alveoli in lung tissues together with reduced alveolar space and decreased alveoli fusion when compared with those treated with oe-NC vector (*p* < 0.05). In addition, BPD was found to be further alleviated in neonatal mice with hyperoxia-induced BPD upon treatment of miR-29a antagomir + oe-GAB1 (*p* < 0.05). Measurement of alveolar chord length, assessment of lung cell proliferation by immunohistochemistry, and detection on lung cell apoptosis by TUNEL staining were conducted to further evaluate the function of GAB1 regulated by miR-29a. As depicted in Fig. 4D-H, K-L, no obvious changes were detected in chord length, positive expression rate of Ki67 and CD31 proteins and cell apoptosis between the mice exposed to RA and those exposed to RA following other treatment (*p* > 0.05). The positive expression rate of Ki67 and CD31 proteins displayed a notable increase in lung tissues of neonatal mice with hyperoxia-induced BPD after being treated with oe-GABI vector with a remarkable decline in alveolar chord length and cell apoptosis compared with those treated with oe-NC vector (*p* < 0.05). These parameters showed a more significant tendency in neonatal mice with hyperoxia-induced BPD after receiving an injection of miR-29a antagomir combined with oe-GAB1 vector (*p* < 0.05). Next, western blot analysis was performed to evaluate the expression of apoptosis-related proteins (Bax, Caspase-3 and Bcl-2). As the results demonstrated, the lung tissues of neonatal mice with hyperoxia-induced BPD after administrating with oe-GAB1 vector had decreased Bax and Caspase-3 expression yet increased Bcl-2 expression compared to those treated with oe-NC vector (*p* < 0.05). Injection of miR-29a antagomir combined with oe-GAB1 vector into neonatal mice with hyperoxia-induced BPD resulted in a more significant tendency in the expression of apoptosis-related proteins (*p* < 0.05; Fig. 4I-J). The data collected indicated that upregulation of GAB1 ameliorated BPD in neonatal mice, which can be further strengthened by silencing miR-29a.

## Discussion

Among the complications associated with mechanical ventilation in preterm infants, BPD is a serious disorder considering the lack of effective treatment options (Yeh et al. 2016). There is compelling evidence highlighting the regulatory role of miRNAs in the pathogenesis of BPD by targeting specific genes; for instance, miR-206 was found to promote cell apoptosis and suppress proliferation in BPD through the reduction of fibronectin 1 expression (Zhang et al. 2013). Therefore, understanding the molecular mechanisms by which the proliferation and apoptosis were regulated in BPD might contribute to offer novel strategies for preventing or arresting progression of this disease. The current study revealed that inhibition of miR-29a could alleviate BPD in neonatal mice by upregulating GAB1.

We initially found that miR-29a expression was increased but GAB1 was downregulated in lung tissues of neonatal mice following hyperoxia-induced BPD modeling, indicating that aberrant expression of miR-29a might be associated with the development of BPD miR-29a involves in both idiopathic pulmonary fibrosis cells and lung cancer cells from bronchoalveolar lavage (Bibaki et al. 2018). Furthermore, in agreement with our findings, a previous study suggested that the expression of miR-29a is enriched in the lung tissues of neonatal mice with hyperoxia-induced BPD (Dong et al. 2012). Based on the results from the bioinformatics analysis and confirmation of dual-luciferase reporter gene assay, GAB1 was found to be a target gene of miR-29a and can be negatively regulated by miR-29a. Similarly, a former conducted study revealed that GAB1 is a target gene of miR-29a (Ruan et al. 2018). Moreover, a previous study showed that inhibition of GAB1 may impair surfactant protein homeostasis, subsequently predisposing mice to lung injuries, which suggests that GAB1 is involved in lung injury defense (Wang et al. 2016). From the results above, it could be concluded that miR-29a may negatively regulate GAB1, and both miR-29a and GAB1 may be involved in BPD.

Importantly, the current study showed that downregulation of miR-29a could repress cell apoptosis and promote cell proliferation, thus preventing the BPD through the upregulation of GAB1, evidenced by increased expression of Bax and Caspase-3 as well as stimulated positive expression rate of Ki67 and CD31 proteins, yet diminished Bcl-2 expression. Bax functions as an apoptosis promoter and Bcl-2 serves as an apoptosis inhibitor, and the Bax/Bcl-2 ratio may upregulate caspase-3 expression, thus modulating apoptosis (Salakou et al. 2007). miR-29a has been reported to promote cell apoptosis since it promotes the expression of caspase-3 and Bax while reducing Bcl-2 expression in cardiomyocytes after myocardial ischemia-reperfusion (Wang et al. 2015). Ki67 is a nuclear antigen associated with cell proliferation and is widely used as a marker of cell proliferation (Alapati et al. 2014). It has been reported to participate in the pulmonary pathological process in ventilated/oxygen treated preterm infants with respiratory distress syndrome (Lu et al. 2012). CD31, a pan-endothelial cell marker, is found to be expressed in both microvascular and larger vascular endothelial cells in alveolar areas (Ghelfi et al. 2011). The protein expression level of CD31 has been detected to be decreased in the BPD group (Wang et al. 2014), a finding of which was similar to ours: the positive expression rate of CD31 protein displayed a notable decline in lung tissues of neonatal mice with hyperoxia-induced BPD. miR-29b leads to retarded growth of intestinal epithelial cells and suppressing GAB1 expression induces apoptosis (An et al. 2016). In addition, decreased miR-29a-3p expression gives rise to enhanced proliferation of glioma cells via the upregulation of GAB1 (Shao et al. 2018). The critical pro-survival role of GAB1 was also indicated in rat neonatal cardiomyocytes as overexpressed GAB1 was found to result in reduction of apoptosis (Cherif et al. 2015). There is increasing evidence displaying that miRNAs regulate translation of messenger RNAs (mRNAs) in a mouse model of hyperoxia-induced BPD. Abnormal lung development is accompanied by significant increases in the levels of multiple miRNAs and corresponding decreases in the levels of predicted mRNA targets, many of which have known or suspected roles in pathways altered in BPD (Dong et al. 2012). Taken together, the results presented in this study conclusively showed that downregulated miR-29a expression could suppress cell apoptosis and induce proliferation of BPD cells via upregulating GAB1.

## Conclusion

**Fig. 5:**
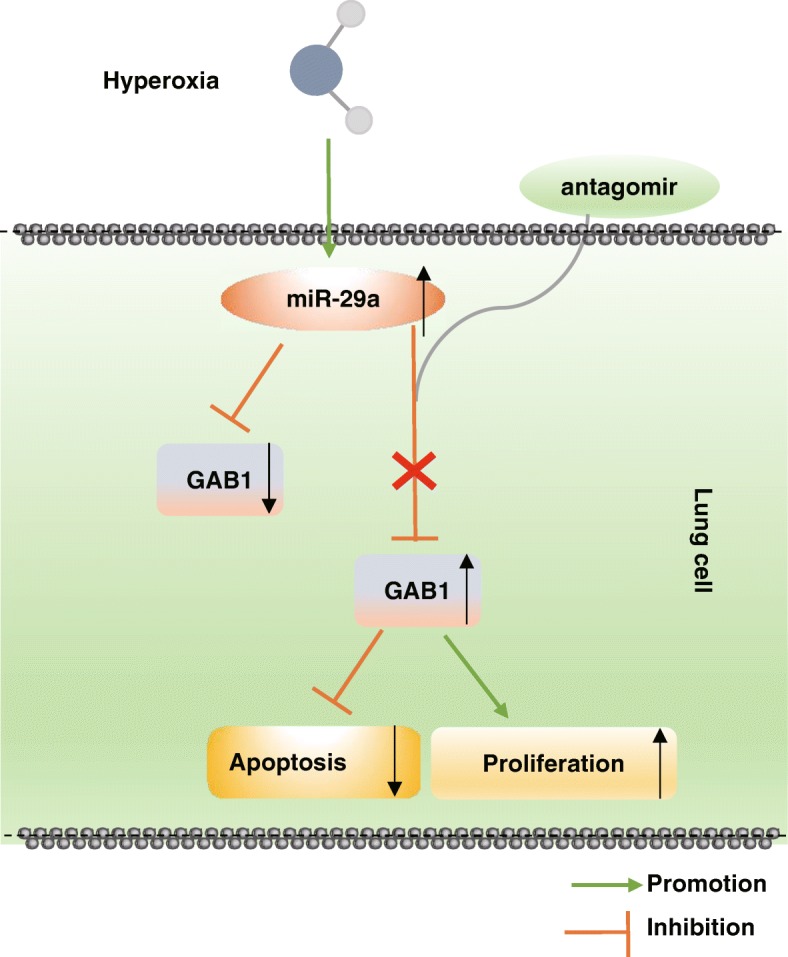
Schematic representation of miR-29a in hyperoxia-induced BPD in neonatal mice. miR-29a is highly expressed and GAB1 is lowly expressed in hyperoxia-induced BPD in neonatal mice. Downregulation of miR-29a attenuates cell apoptosis and promotes cell proliferation in lung tissues by increasing GAB1 expression, ultimately retarding the development of BPD. miR-29a, microRNA-29a; GAB1, GRB2-associated-binding protein 1; BPD, bronchopulmonary dysplasia

Overall, our findings support that downregulation of miR-29a could potentially help prevent BPD by upregulating its target GAB1 gene (Fig. 5). This provides a new insight into the role and molecular mechanism of miR-29a in BPD. Therefore, miR-29a could be used as a potential therapeutic marker for BPD treatment. Nevertheless, the findings provided in this study are preliminary, indicating more studies in this area are required in the future to investigate the detailed mechanisms of miR-29a in BPD.

## Data Availability

The datasets generated during the current study are available.

## Abbreviation

3’UTR: 3’untranslated region
A/S: alveolar area/pulmonary septal area ratio
ANOVA: analysis of variance
ATCC: American Type Culture Collection
Bax: Bcl-2-associated X protein
BCA: bicinchoninic acid
Bcl-2: B-cell lymphoma 2
BPD: bronchopulmonary dysplasia
cDNA: complementary DNA
ECL: enhanced chemiluminescence
GAB1: GRB2-associated-binding protein 1
GAPDH: glyceraldehyde 3-phosphate dehydrogenase
HE: hematoxylin-eosin
IgG: immunoglobulin G; DAB diaminobenzidine
KM: Kunming
miR-29a: microRNA-29a
miRNAs: microRNAs
Mut: mutant
NC: negative control
NSCLC: non-small cell lung cancer
oe: overexpressed
RA: room air
RAC: radial alveolar counts
RT-qPCR: reverse-transcription quantitative polymerase chain reaction
SDS-PAGE: sodium dodecyl sulfate-polyacrylamide gel electrophoresis
TBST: Tris-buffered saline Tween-20
TUNEL: TdT-mediated dUTP-biotin nick end-labeling
Wt: wild type
